# Public knowledge, attitudes, and practices towards herbal medicines; a cross-sectional study in Western Saudi Arabia

**DOI:** 10.1186/s12906-022-03783-y

**Published:** 2022-12-08

**Authors:** Syed Faisal Zaidi, Sheikh Abdul Saeed, Muhammad Anwar Khan, Aslam Khan, Yaqoub Hazazi, Mohammed Otayn, Mohammed Rabah, Muhammad Daniyal

**Affiliations:** 1 Faculty of Eastern Medicine, Hamdard University, Islamabad Campus, Islamabad, 44000 Pakistan; 2 College of Medicine, King Saud bin Abdulaziz University for Health Sciences, Jeddah, Saudi Arabia; 3 Department of Physiology, Bahria University Medical & Dental College, Karachi, Pakistan; 4 King Abdullah International Medical Research Center, King Abdulaziz Medical City, Jeddah, Saudi Arabia; 5 Riphah Institute of Pharmaceutical Sciences, Riphah International University, Raiwind Road, Lahore, Punjab 54000 Pakistan; 6 TCM & Ethnomedicine Innovative & Development International Laboratory, School of Pharmacy, Hunan University of Chinese Medicine, Changsha, Hunan 410208 People’s Republic of China

**Keywords:** Herbal medicine knowledge, Attitude, Practice, General public awareness, Jeddah, Western Saudi Arabia

## Abstract

**Background:**

Herbal medicines, derived from medicinal plants, are among the most popular alternative remedies around the globe. In Saudi Arabia, herbal medicines are extensively used by public as part of the culture as well as religious norms. Therefore, this study aimed to evaluate knowledge, attitudes, and practices regarding herbal medicines among the general population in Saudi Arabia.

**Methods:**

A descriptive cross-sectional survey study was conducted on the general population of Jeddah city with the help of a validated and self-administered questionnaire. Sample size was calculated to be 450 with subjects between 20 and 60 years of either gender. Descriptive and inferential statistical analysis was performed using SPSS.

**Results:**

Results of our data showed that 173 (42.2%) of the total participants used herbal medicines, however, significant association was found between female gender and the usage of herbal medicines (*p* < 0.001). Approximately, more than half (56.0%) of the respondents agreed that herbal medicines could be used to promote health and treat illnesses, and 45% respondents agreed that herbal medicines are safe. 153 (37.4%) of the participants opined that herbal medicines could be taken with conventional or allopathic medicine. The data also showed a significant (*p* < 0.05) association between knowledge about the source of herbal medicine and gender where females were found to have more knowledge compared to males. Moreover, a significantly higher number of chronic disease patients were using herbal medicines compared to individuals with no medical problems (*p* < 0.001). Strikingly, majority (*n* = 204; 49.9%) of the respondents used herbal medicines as a first choice when sick, while a good number (*n* = 172; 42.1%) of respondents did not consult doctors before taking herbal medicines.

**Conclusion:**

The use of herbal medicines is common among the general population of Jeddah. Although most of the participants believed that the herbal medicines are safe and do not require consultation, there is a dire need to increase awareness as well as to establish effective strategies to evaluate the safety, efficacy and quality of the herbal medicines for propitious consequences of this commonly used entity in the local society.

**Supplementary Information:**

The online version contains supplementary material available at 10.1186/s12906-022-03783-y.

## Introduction

Herbal medicine is the use of plant parts such as roots, leaves, flowers, bark, berries, and seeds to treat or prevent disease. Devil’s claw, kava, Echinacea, ginseng, ginger, St. John’s wort, black and blue cohosh, red raspberry leaf, and castor oil are among the most commonly used medicinal herbs [1]. For a long time, herbal remedies have been used to prevent and treat a variety of health problems around the world [2]. Herbal medications have a lengthy history of being used in the production of drugs such as aspirin, morphine, and digoxin, among others. Herbal medications are used to treat a variety of problems, such as microbial and viral infections, reproductive health difficulties, mental and immunological disorders, and noncommunicable diseases such as malaria, cancer etc. [1].

Herbal medicines, as a part of complementary therapies, are used worldwide as self-medication and also prescribed by some general practitioners [3]. World Health Organization (WHO) defines herbal medicine as plant-derived material and products which have therapeutic and other positive effects on human health and contain raw or processed ingredients from one plant or a combination of different plants [4]. Many research studies carried out on herbal medicines have proved scientifically that they are effective for the treatment of different types of clinical disorders [5]. In the developing countries, more than 80% of people use herbal medicine as the primary mode of treatment for their illnesses [6]. In the Arab world, use of herbal medicine is very common, probably due to the belief that all the herbal preparations are safe. However, this perception is not really true [7]. Nowadays, these types of behavior are also noted in general practitioners who are prescribing the herbal medicine for the treatment of common illnesses [8]. Herbal medicines are included in the category of over-the-counter (OTC) drugs, that is why they are easily available through pharmacies. Many people use herbal medicine alone or in combination with other conventional medicine without informing the physician and it may lead to serious illness [9]. Plants have been used for medicinal purposes since ancient times and form the basis of many modern day medicines. Several conventional drugs can be traced back to plant sources, for example, aspirin (from willow bark), digoxin (from foxglove), quinine (from cinchona bark), and morphine (from the opium poppy). Still, drug companies continue to tap this natural treasure and are engaged in the large-scale pharmacologic screening of herbs [10]. In developed countries, herbal medicine has been coined under the category of Complementary or Alternative medicine (CAM).

In the past few decades, there has been renewed attention and interest in the use of traditional medicine globally. Hence, herbal medicine has played an important role in the treatment of several acute and chronic diseases. In Africa, 80% of the populations somehow employ traditional herbal medicine for various illnesses and eventually the annual growth of herbal products market worldover has crossed US$ 60 billion [11]. On the other side, traditional medicine has also been used in the treatment and care of such life-threatening illnesses like malaria and AIDS. In Ghana, Mali, Nigeria and Zambia, herbal medicines are the first-line treatment for more than 60% of children with a high fever. Studies in Africa and North America have shown that up to 75% of people living with HIV/AIDS either use traditional medicine only or use it in combination with other medicines for various symptoms or conditions [11].

Previous studies on herbal medicines use in Saudi Arabia were mainly focused on specific groups of population like pregnant ladies or patients with illnesses such as hypertension, diabetes mellitus, ischemic heart disease, end-stage renal diseases, and allergic disorders [12–14]. One study has also addressed the physicians’ concerns and awareness of the use of herbal medicine by their patients [15]. Most of these studies were conducted in Riyadh or the central region of Saudi Arabia. However, to the best of our knowledge, no study so far has documented general public’s perception with respect to herbal medicines in the Western part of Saudi Arabia. Therefore, the aim of this study was to explore knowledge, attitude and practices regarding herbal medicines among the general public of Jeddah, which is the biggest cosmopolitan city in Western Saudi Arabia and the second largest city in the Kingdom.

## Methodology

### Study design and sampling

A descriptive cross-sectional survey study was carried out with the questionnaire. It was conducted on the general public living in Jeddah, from August 2018 to September 2018. Sample size was calculated by Raosoft® website (http://www.raosoft.com/samplesize.html). The required sample size was estimated at 95% confidence level with an estimated 50% response distribution and a margin of error of ±5%. The required minimum sample size was determined to be 384; the final sample size taken was 460 to account for a 20% non-response rate. Non-probability convenience sampling technique was used for selecting the sample. Research teams were present at the site of the public places and participants were invited to take part in the study. Participation in the study was completely voluntary and no incentives were given to the participants. There was no eligibility requirement for subjects to be included in the research; the questionnaire was distributed equally among all population who were ready to participate in the study. Subjects were between 20 and 60 years of age from either gender.

### Development and validation of the questionnaire

A questionnaire was created after extensive survey of literature and a pilot test was conducted on 30 volunteers from the general public and Cronbach’s alpha was calculated, which yielded a high reliability of 0.81–0.85 (knowledge, attitude and practice). The content validity was done by inviting 2 independent subject experts and few modifications were done after the feedback. The questionnaire consisted of three sections (knowledge, attitude, and practice) and five parts. The first part included 14 items concerned with information about the demographic characteristics of the respondents. Part two consisted of 3 questions to determine the usage of herbal medicines. Part three included 10 statements to determine the knowledge of herbal medicines using a three points Likert scale (Yes, No, Not sure). Part four included eight-point statements about the attitude towards herbal medicines that were measured using a five points Likert scale (Strongly agree, Agree, Neutral, Disagree, and Strongly disagree). The last part included eight statements to determine practices towards herbal medicines. The responses in this section were measured using a five points Likert scale (Strongly agree, Agree, Neutral, Disagree and Strongly disagree). The questionnaire was translated into Arabic and subjected to a process of forward and backward translation. The questionnaires, along with a written consent form, were disseminated among the general population which described the purpose of the study as well as assured them of confidentiality. After the distribution, the participants were allowed to complete the questionnaire, while one of the investigators was available to answer their queries (if any).

### Study approval

The study was approved by the ethical committee of the University, Institutional Review Board (IRB), King Abdullah International Medical Research Center (KAIMRC), Ministry of National Guard-Health Affairs (MNG-HA), with study number SP17/385/J and Memo. Ref. No. IRBC/1521/17, while written informed consent was taken from each participant before the study.

### Statistical analysis

Data analysis was executed using IBM Statistic SPSS (SPSS Inc., Chicago, IL, USA) version 20.0., where qualitative variables were presented as frequencies and percentages and analysis was performed by using Chi-square and Fisher-Exact tests. The level of statistical significance was set at *p* < 0.05.

## Results

### Study population

Table 1 shows the demographic characteristics of the study population. Out of 450 individuals who were approached, 28 refused to participate and 13 incomplete questionnaires were excluded. As a result, 409 completed questionnaires were included in the study giving a response rate of 90.8%. Mean age of participants was 35.3 ± 10.80 (mean ± SD) years. The highest number of participants was Saudis (*n* = 265; 64.8%), followed by Egyptians (*n* = 44; 10.8%), Pakistanis (*n* = 18; 4.4%), Jordanians (*n* = 16; 3.9%), and other nationalities (*n* = 63; 15.4%). Majority of participants (*n* = 262; 64.1%) were males, and most of them were married (*n* = 204; 49.9%). Regarding the educational level, 63.4% (*n* = 149) of respondents had attained secondary school education and 29.3% (*n* = 120) were either enrolled or had completed university. Most of the participants (*n* = 173; 42.3%) were paid employees. Break-up of rest of them is as follows: 22.0% (*n* = 90) unemployed, 15.4% (*n* = 63) housewives, 10.3% (*n* = 42) self-employed and 10% (*n* = 41) retired. Over 50% (*n* = 234; 57.2%) of the studied population had salary less than 3000 SR/month, and only (*n* = 62; 15.2%) were healthcare workers. The personal health status of 72.4% (*n* = 296) participants was excellent, 16.4% (*n* = 67) very good, 8.6% (*n* = 35) good, 2.0% (*n* = 8) fair, and 0.7% (*n* = 3) poor. Two hundred and six (50.37%) individuals reported to have chronic diseases such as hypertension (n 46; 11.2%), diabetes (*n* = 62; 15.2%), heart disease (*n* = 26; 6.4%), respiratory (*n* = 31; 7.6%), kidney disease (np =14; 3.4%), and joint diseases (*n* = 27; 6.6%).

**Table 1:** Distribution of the studied population according to sociodemographic characteristics

Socio-demographic Characteristics (***n*** = 409)	n (%)
**Gender**	
Male	262 (64.1)
Female	147 (35.9)
**Ethnicity**	
Saudi	265 (64.8)
Egyptian	44 (10.8)
Jordanian	16 (3.9)
Pakistani	18 (4.4)
Other	63 (15.4)
**Marital Status**	
Single	143 (35.0)
Married	204 (49.9)
Widowed	29 (7.1)
Divorced	27 (6.6)
Separated	5 (1.2)
**Educational Status**	
None	19 (4.6)
Primary School	63 (15.4)
Secondary School	149 (36.4)
College	58 (14.2)
University	120 (29.3)
**Employment Status**	
Employed for wags	173 (42.3)
Self-employed	42 (10.3)
Housewife	63 (15.4)
Retired	41 (10.0)
Unemployed	90 (22.0)
**Monthly Income (SAR)**	
< 3000	234 (57.2)
3000–4999	84 (20.5)
5000–7999	63 (15.4)
8000–10,000	15 (3.7)
> 10,000	13 (3.2)
**Occupation related to healthcare**	
No	347 (84.8)
Yes	62 (15.2)
**Family members occupation related to healthcare**	
No	273 (66.7)
Yes	136 (33.3)
**Cigarette Smoking**	
No	252 (61.6)
Yes	157 (38.4)
**Shisha Smoking**	
No	288 (70.4)
Yes	121 (29.6)
**Respiratory illness**	
No	378 (92.4)
Yes	31 (7.6)
**Diabetes Mellitus**	
No	347 (84.8)
Yes	62 (15.2)
**Hypertension**	
No	363 (88.8)
Yes	46 (11.2)
**Heart problems**	
No	383 (93.6)
Yes	26 (6.4)
**Kidney diseases**	
No	395 (96.6)
Yes	14 (3.4)
**Joint diseases**	
No	382 (93.4)
Yes	27 (6.6)
**Personal health status**	
Poor	3 (0.7)
Fair	8 (2.0)
Good	35 (8.6)
Very good	67 (16.4)
Excellent	296 (72.4)

*SAR* Saudi Arab Riyal equal to 0.27 USD

### Use of herbal medicines

Table 2 shows the use of herbal medicines according to differences by the study population characteristics. Herbal medicines were used by one hundred and seventy-three (42.29%; 95% CI: 1.44–3.27) respondents and were found to be significantly common among females compared to males (*p* < 0.001). Regarding ethnicity, the usage of herbal medicines was found to be significantly common among other nationalities (95% CI: 1.08–1.25; *p* = 0.003). Also, separated people were found to be using herbal medicines more commonly than single, married, widowed, and divorced (95% CI: 1.08–1.25) with *p*-value 0.003. Self-employed individuals and housewives were found to be using herbal medicines more frequently (48.54%; 95% CI: 2.37–2.60) than others, with *p*-value < 0.001. There was no significant association between the usage of herbal medicines and the monthly income of participants (*p* = 0.133).

**Table 2:** Association between the use of herbal medicines and respondent’s characteristics

Characteristics		Frequency (%)			OR (95% CI)	*p*-value
		Yes	No	Total		
**Gender**	Male	93 (35.5)	169 (64.5)	262	2.17 (1.44–3.27)	< 0.001
	Female	80 (54.4)	67 (45.6)	147		
**Ethnicity**	Saudi	97 (36.6)	168 (63.4)	265	(1.08–1.25)	0.003
	Egyptian	19 (43.2)	25 (56.8)	44		
	Jordanian	7 (43.8)	9 (56.3)	16		
	Pakistani	11 (61.1)	7 (38.9)	18		
	Other	39 (61.9)	24 (38.1)	63		
**Marital status**	Single	35 (24.5)	108 (75.5)	143	(1.81–1.98)	< 0.001
	Married	104 (51.0)	100 (49.0)	204		
	Widowed	12 (41.4)	17 (58.6)	29		
	Divorced	17 (63.0)	10 (37.0)	27		
	Separated	4 (80)	1 (20)	5		
**Employment status**	Employed for wages	75 (43.4)	98 (56.6)	173	(2.37–2.60)	< 0.001
	Self employed	26 (61.9)	16 (38.1)	42		
	Housewife	39 (61.9)	24 (38.1)	63		
	Retired	14 (34.1)	27 (65.9)	41		
	Unemployed	19 (21.1)	71 (78.9)	90		
**Income (SAR)**	< 3000	90 (38.5)	144 (61.5)	234	(1.65–1.86)	0.133
	3000–4999	34 (40.5)	50 (59.5)	84		
	5000–7999	34 (54.0)	29 (46.0)	63		
	8000–10,000	9 (60.0)	6 (40.0)	15		
	> 10,000	6 (46.2)	7 (53.8)	13		
**Respiratory disease**	No	152 (40.2)	226 (59.8)	378	3.122 (1.43–6.815)	0.003
	Yes	21 (67.7)	10 (32.3)	31		
**Diabetes Mellitus**	No	134 (38.6)	213 (61.4)	347	2.695 (1541–4.713)	< 0.001
	Yes	39 (62.9)	23 (37.1)	62		
**Hypertension**	No	139 (38.3)	224 (61.7)	363	4.566 (2.287–9.115)	< 0.001
	Yes	34 (73.9)	12 (26.1)	46		
**Heart disease**	No	156 (40.7)	227 (59.3)	383	2.749 (1.195–6.324)	0.014
	Yes	17 (65.4)	9 (34.6)	26		
**Kidney disease**	No	162 (41.0)	233 (59.0)	39	5.274 (1.448–19.201)	0.005
	Yes	11 (78.6)	3 (21.4)	14		
**Joint disease**	No	154 (40.3)	228 (59.7)	382	3.515 (1.501–8.235)	0.002
	Yes	19 (70.4)	8 (29.6)	27		
**No medical problems**	No	88 (59.5)	60 (40.5)	148	0.329 (0.217–0.5)	< 0.001
	Yes	85 (32.6)	176 (67.4)	261		
**Visit store**	None	31 (14.2)	187 (85.8)	218	(0.63–0.8)	< 0.001
	1–4 times	77 (70.0)	33 (30.0)	110		
	5–10 times	43 (75.4)	14 (24.6)	57		
	> 10 times	22 (91.7)	2 (8.3)	24		
**Personal health**	Excellent	108 (36.5)	188 (63.5)	296	(4.5–4.65)	0.003
	Very good	36 (53.7)	31 (46.3)	67		
	Good	22 (62.9)	13 (37.1)	35		
	Fair	5 (62.5)	3 (37.5)	8		
	Poor	2 (66.7)	1 (33.3)	3		

*SAR* Saudi Arab Riyal equal to 0.27 USD

Out of 173 participants, who were reported to be using herbal medicines, 141 (81.5%) had chronic diseases such as; respiratory disease (*n* = 21; 67.7%; 95% CI: 1.43–6.815), diabetes mellitus (*n* = 39; 62.9%; 95% CI: 1541–4.713), hypertension (*n* = 34; 73.9%; 95% CI: 2.287–9.115), heart disease (n-17; 65.4%; 95% CI: 1.195–6.324), kidney disease (*n* = 11; 78.6%; 95% CI: 1.448–19.201) and joint disease (*n* = 19; 70.4%; 95% CI: 1.501–8.235). *P*-values were (0.003), (< 0.001), (< 0.001), (0.014), (0.005) and (0.002), respectively. This shows that a significantly higher number of the chronic disease patients were using herbal medicines, whereas individuals with no medical problems were least commonly using herbs (*n* = 85; 32.6%; 95% CI: 0.217–0.5), *p*-value (< 0.001).

Results showed that people who visited herbal stores more than 10 times in the past 6 months (*n* = 22; 91.7%) were significantly using herbs more commonly compared to those who visited stores 5–10 times, 1–4 times, and those who did not visit stores before (95% CI: 0.63–0.8; *p*-value < 0.001). Also, herbs were commonly used by those with poor personal health status (*n* = 2; 66.7%) in comparison to people with excellent (*n* = 108; 36.5%), very good (*n* = 36; 53.7%), good (n = 22; 62.9%), and fair (*n* = 5; 62.5%) personal health status (95% CI: 4.5–4.65; *p* = 0.003).

### Knowledge regarding herbal medicines

**Fig. 1:**
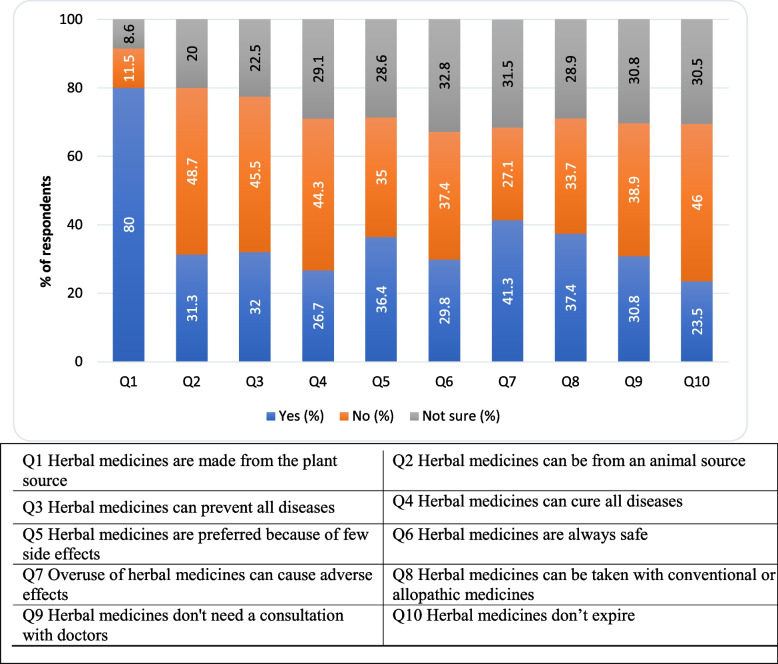
The percentages of participants knwoledge about herbal medicines

Figure 1 shows the respondents’ knowledge of herbal medicines. Respondents generally said yes to the following statements**:** i**)** Herbal medicines are made from plant source (*n* = 327; 80%), ii) Herbal medicines are preferred because of few side effects (*n* = 149; 36.4%), iii) Overuse of herbal medicines can cause adverse effects (*n* = 169; 41.3%), iv) Herbal medicines can be taken with conventional or allopathic medicines (*n* = 153; 37.4%). Respondents generally said no to the following statements**:** i) Herbal medicines can be from animal sources (*n* = 199; 48.7%), however, 128 (31.3%) were certain that herbal medicines can come from the animal sources, ii) Herbal medicines can prevent all diseases (*n* = 186; 45.5%), iii) Herbal medicines can cure all diseases (*n* = 181; 44.3%), iv) Herbal medicines are always safe (*n* = 153; 37.4%), v) Herbal medicines don’t need consultation with doctors (*n* = 159; 38.9%), however, a good number (*n* = 126; 30.8%) of respondents believed in no consultation with doctors, vi) Herbal medicines don’t expire (*n* = 188; 46.0%).

### Attitude towards herbal medicines

**Fig. 2:**
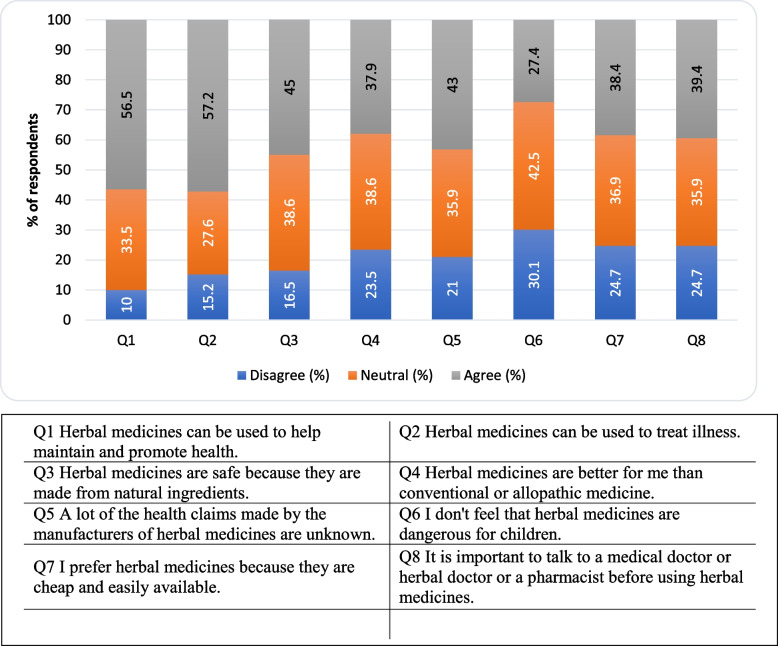
The percentages of participants attitude towards herbal medicines

Figure 2 shows the respondents’ attitude toward medicines. Respondents generally agreed with the following statements: i) Herbal medicines can be used to help maintain and promote health (*n* = 231; 56.5%), ii) Herbal medicines can be used to treat illness (*n* = 234; 57.2%), iii) Herbal medicines are safe because they are made from natural ingredients (*n* = 183; 45.0%), iv) A lot of the health claims made by the manufacturers of herbal medicines are unknown (*n* = 176; 43.0%), v) I prefer herbal medicines because they are cheap and easily available (*n* = 157; 38.4%) and vi) It is important to talk to a medical doctor or herbal doctor or a pharmacist before using herbal medicines (*n* = 161; 39.4%). Respondents generally disagreed with the following statements: i) Herbal medicines are better for me than conventional or allopathic medicine (*n* = 158; 38.6%), ii) I don’t feel that herbal medicines are dangerous for children (*n* = 174; 42.5%).

### Practice related to herbal medicines

**Fig. 3:**
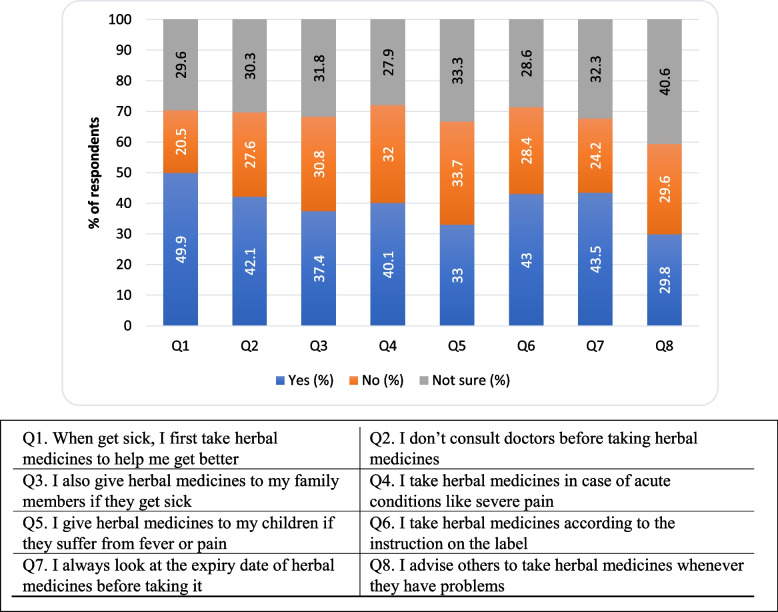
The percentages of participants practice towards herbal medicines

Figure 3 demonstrates the respondents’ practice toward herbal medicines. Respondents generally said yes to the following statements: i) When get sick, I first take herbal medicines to help me get better (*n* = 204; 49.9%), ii) I don’t consult doctors before taking herbal medicines (*n* = 172; 42.1%), iii) I also give herbal medicines to my family members if they get sick (*n* = 153; 37.4%), iv) I take herbal medicines in case of acute conditions like severe pain (*n* = 164; 40.1%), v) I take herbal medicines according to the instructions on the label (*n* = 176; 43%) and vi) I always look at the expiry date of herbal medicines before taking them (*n* = 178; 43.5%). Interestingly, almost equal number of respondents said yes or no to the following statements: i) I give herbal medicines to my children if they suffer from fever or pain (yes: *n* = 135; 33.0%, no: *n* = 138; 33.7%), ii) I advise others to take herbal medicines whenever they have problems (yes: *n* = 122; 29.8%, no: *n* = 121; 29.6%).

### Association of demographics with the knowledge of participants towards herbal medicine

Table 3 summarizes the association of demographics with knowledge of respondents towards herbal medicine. This table includes only those responses which are statistically different. A significantly higher number of respondents (84.6%) agreed with statement no.3 i.e. “Herbal medicines are preferred because of less side effects” with an increase in income level. Similarly, the percentage of response to statement no. 5 that is “Herbal medicines are preferred because of less side effects” and no.7 that is “Overuse of herbal medicines can cause adverse effects” is also significantly higher with an increase in educational status. 47.5% of the respondents with a university degree agree with statement no. 3, whereas 50.8% of such respondents agree with statement no. 5. This shows that with an increase in educational status, there is an increase in the the knowledge about herbal medicine as well. Gender difference did not show any significant difference to the knowledge statements except no. 9, that is “herbal medicine does not need consultation with doctors”. A significantly higher number of male respondents (91) agree with statement no.9 as compared to female respondents (35).

**Table 3:** Association of income, educational status and gender with knowledge of herbal medicines

Association of Demographic with Knowledge			Not sure	No	Yes	***p***-value
Herbal medicines can prevent all diseases	Income (SAR)	< 5000	68 (21.4)	158 (49.7)	92 (28.9)	< 0.001**
		5000-10,000	24 (30.8)	26 (33.3)	28 (35.9)	
		> 10,000	0 (0.0)	2 (15.4)	11 (84.6)	
		**Total**	**92**	**186**	**131**	
Herbal medicines are preferred because of less side effects	Educational Status	None	7 (36.8)	6 (31.6)	6 (31.6)	0.016*
		Primary School	19 (30.2)	25 (39.7)	19 (30.2)	
		Secondary School	34 (22.8)	61 (40.9)	54 (36.2)	
		College	23 (39.7)	22 (37.9)	13 (22.4)	
		University	34 (28.3)	29 (24.2)	57 (47.5)	
		**Total**	**117**	**143**	**149**	
Overuse of herbal medicines can cause adverse effects	Educational Status	None	9 (47.4)	6 (31.6)	4 (21.1)	0.047*
		Primary School	24 (38.1)	19 (30.2)	20 (31.7)	
		Secondary School	45 (30.2)	49 (32.9)	55 (36.9)	
		College	18 (31.0)	11 (19.0)	29 (50.0)	
		University	33 (27.5)	26 (21.7)	61 (50.8)	
		**Total**	**129**	**111**	**169**	
Herbal medicines don’t need consultation with doctors	Gender	Male	70 (26.7)	101 (38.5)	91 (34.7)	0.034*
		Female	54 (36.7)	58 (39.5)	35 (23.8)	
		**Total**	**124**	**159**	**126**	

*chi-squared test

**fisher’s exact test

*SAR* Saudi Arab Riyal equal to 0.27 USD

### Association of demographics with the attitude of participants towards herbal medicine

Table 4 summarizes the association of educational status and income with the attitude of the respondent towards herbal medicines. In this table, only those responses are included, which show a statistical difference. It can be seen from the results that the response of the respondents is significantly (*p* < 0.006) increasing with education status of the respondents for statement no.1 i.e. “Herbal medicines can be used to help maintain and promote health”. People with a higher degree have agreed with statement no.1 more as compared to those with a lower or zero educational status. Similarly, the response of people to statement no.1 and also no.3 i.e. “Herbal medicines are safe because they are made from natural ingredients” is seen to increase significantly with an increase in income level. 100 and 84.6% of the respondents having a monthly income of more than SR. 10,000, agree with statements no.1 and 3, respectively.

**Table 4:** Association of educational status and income with attitude towards herbal medicine

Association of Demographics with Attitude			Disagree	Neutral	Agree	***p***-value
Herbal medicines can be used to help maintain and promote health	Educational Status	None	0	11 (57.9)	8 (42.1)	0.006*
		Primary School	7 (11.1)	19 (30.2)	37 (58.7)	
		Secondary School	17 (11.4)	56 (37.6)	76 (51.0)	
		College	6 (10.3)	26 (44.8)	26 (44.8)	
		University	11 (9.2)	25 (20.8)	84 (70.0)	
		**Total**	**41**	**137**	**231**	
Herbal medicines can be used to help maintain and promote health	Income	< 5000	29 (9.1)	114 (35.8)	175 (55.0)	0.007**
		5000-10,000	12 (15.4)	23 (29.5)	43 (55.1)	
		> 10,000	0	0	13 (100)	
		**Total**	**41**	**137**	**231**	
Herbal medicines are safe because they are made from natural ingredients	Income	< 5000	53 (16.7)	130 (41.0)	134 (42.3)	0.025*
		5000-10,000	13 (16.7)	27 (34.6)	38 (48.7)	
		> 10,000	2 (15.4)	0	11 (84.6)	
		**Total**	**68**	**157**	**183**	

*chi-squared test

**fisher’s exact test

### Association of demographics with the practices of participants towards herbal medicine

Table 5 summarizes the association demographics with practices or use of respondent towards herbal medicine. Most of the demographic characteristics of the respondent did not show any significant effect on the practice/use of herbal medicine (Data not shown) except for the educational status and gender, which showed significant effect toward statement no. 6 i.e. “I take herbal medicines according to the instruction on the label” and no.7 i.e. “I always look at the expiry date of herbal medicines before taking it”. It was also found that 63 (52.5%) of the respondents having a university degree, used the herbal medicines according to the instructions on the label (no. 6). Similarly, 73 (27.9%) of the male and 26 (17.7%) female did not look at the expiry date of herbal medicines before taking them (no.7), while 112 (42.7%) male and 66 (44.9%) female respondent did look it.

**Table 5:** Association of educational status and gender with practices of herbal medicine

Association of Demographic with Practice			Not sure	No	Yes	***p***-value
I take herbal medicines according to the instruction on the label	Educational Status	None	2 (10.5)	12 (63.2)	5 (26.3)	0.003
		Primary School	19 (30.2)	25 (39.7)	19 (30.2)	
		Secondary School	46 (30.9)	41 (27.5)	62 (41.6)	
		College	18 (31.0)	13 (22.4)	27 (46.6)	
		University	32 (26.7)	25 (20.8)	63 (52.5)	
		**Total**	**117**	**116**	**176**	
I always look at the expiry date of herbal medicines before taking it	Gender	Male	77 (29.4)	73 (27.9)	112 (42.7)	0.050
		Female	55 (37.4)	26 (17.7)	66 (44.9)	
		**Total**	**132**	**99**	**178**	

chi-squared test

### Association of age with knowledge and practices of herbal medicine

Table 6 summarizes the association of age with knowledge and practices/use of respondents towards herbal medicine. 181 respondents responded “No”, while 109 responded “Yes” towards the statements “herbal medicines are preferred because of few side effects (knowledge statement no.5)”, with mean age of 34.4 + 10.33 and 38.0 + 11.33 respectively. 153 respondents responded “No”, while 122 responded “Yes” towards the statements “Overuse of herbal medicines can cause adverse effects (knowledge statement no.7)” with mean age of 36.2 + 11.24 and 36.8 + 11.48 respectively. Regarding the practices, only one statement, “I give herbal medicines to my children if they suffer from fever or pain” (practice statement no.5), showed significant difference with age of the respondents. 131 respondents responded “No”, while 164 responded “Yes” towards the statements P5, with mean age of 33.9 + 10.31 and 37.0 + 11.37 respectively.

**Table 6:** Association of age with knowledge and practices of herbal medicine

						95% CI			
		N	Mean	**+**	Std. Dev	Lower Bound	Upper Bound	F	***p***-value
Herbal medicines are preferred because of less side effects (knowledge related statement)	Not sure	119	34.3	+	10.71	32.40	36.29	4.39	0.013
	No	181	34.4	+	10.33	32.93	35.96		
	Yes	109	38.0	+	11.33	35.80	40.11		
Overuse of herbal medicines can cause adverse effects (knowledge related statement)	Not sure	134	33.1	+	9.26	31.48	34.65	4.63	0.010
	No	153	36.2	+	11.24	34.41	38.00		
	Yes	122	36.8	+	11.48	34.73	38.84		
I give herbal medicines to my children if they suffer from fever or pain (practice related statement)	Not sure	114	34.6	+	10.27	32.68	36.49	3.42	0.034
	No	131	33.9	+	10.31	32.14	35.71		
	Yes	164	37.0	+	11.37	35.26	38.77		

ANOVA test

## Discussion

This study found that 173 (42.2%) out of the total participants took herbal medicines and a significant link between female and herbal medicines usage was detected (*p* 0.001). More than one-half (56%) felt that herbal medicines might be used to improve health and treat diseases, while 45% believed that herbal medicines were safe. 153 of the participants (37.4%) believed that a combination of traditional or allopathic medication could be employed. The results also showed a significant association (*p* 0.05) between herbal medicine source knowledge and gender with women having higher information than men. Moreover, participants with chronic diseases take more herbal medication than people without illnesses (*p* 0.001). Surprisingly, most (*n* = 204; 49.9%) of the respondents used herbal remedies as their first choice when they fell unwell, whereas a significant number (*n* = 172; 42.1%), before using herbal medicine, had not seen doctors.

This is the first study regarding the knowledge, attitude, and practices on herbal medicines in the general population of Jeddah, the second largest city of Saudi Arabia. As everywhere else in the world, herbal medicines are used extensively in Saudi Arabia and the findings of our study would help to provide an insight into people’s behavior, attitude and knowledge and exhibit the level of understanding regarding herbal medicines particularly. Most of the previous research has been carried out on patients or specific population in Saudi Arabia, however, very little is documented about herbal medicines knowledge and attitudes among the general public. In the present study, utilization of herbal medicines among adults of Jeddah is found to be 42%. Several international studies have documented results of herbal medicines’ usage in general population. Findings of this study were also consistent with data obtained in other countries. Prevalence of use of herbal medicines was reported as 33.9% in Malaysian adults; in Czech adults as 56.6%; in American population as 57.3%; in Arabian population it was 77.6% [16–19].

A survey done among 1300 individuals documented that 1226 (94%) of the participants utilised herbal medicines for therapeutic purposes. The majority of them, 699 (57%), used herbal medicines on the basis of traditional beliefs or family recommendations 417 (34%). Young respondents < 35 years old who live in cities reveal that they know far more than their counterparts about the use of herbal medicines and the related hazards (*p* < 0.001). Despite the significant frequency of reported side effects, 702 (54%) utilise herbal medicines as their first line of therapy. However, the reasons for the use of herbal medicinal products are believed to be safer, more efficient and cheaper than mainstream medicinal products [20]. Furthermore, the tradition and culture in Saudi Arabia reinforce the use of herbal medicines. In addition, a recent study by Kennedy et al. stated that the majority of pregnant women use herbal medicine due to the perception of herbal medicines as safe therapy [21]. In fact, the accessibility and easiness of herbal medicines subsidize to the amplified use and many people have faith in that traditional recipes are not injurious and do not have side effects [21].

In this study, use of herbal medicines in general population is comparatively lower (42%) than some of the other studies in the world, e.g. in Kuwait (71%) or Nigeria (67%) [22, 23]. The second major finding was identification of the factors which influence the use of herbal medicine like gender, marital status, chronic conditions, medication use and general health perception. Gender differences may partly be explained by the disparity in health needs and social factors such as beliefs and attitudes. Our study finding is consistent with the findings of various other studies that women were more likely to use herbal medicines than men [16, 17, 24, 25]. This was similar in previous studies which could be related to many factors such as that women are more prone to exaggerate the efficacy of herbs and encourage each other to use them [22]. This study shows a notable relation between herbal medicines usage and joint diseases. This may be due to the fact that natural remedies are mostly for topical application like herbal oils and extracts etc. and help in avoiding systemic side effects of such remedies . One study in 2007 based on the survey of 1 year, concluded that almost 4 out of 10 American adults-use CAM i.e., 17.7% [26]. Another study in UK stated that the use of herbal medicines was 41.1% in 1 year [27]. Moreover, a cross-sectional study in UAE stated that 76% of respondents used herbal medicines, with 38% of them using a single herb [19]. Our study outcomes are supportive of the previous studies. One of the reason that people consume herbal medicines might be because of dissatisfaction with conventional medicine [28].

With respect to knowledge, the findings of our study indicated that most of the participants (80%) agreed that herbal medicines are made from plant sources, which gives a hint about how much people were aware when they were asked about herbal medicines in general. One third of participants (37%) acknowledged that herbal medicines could be taken with conventional or allopathic medicines, while in another study conducted in USA, the percentage was higher (55%) [26]. This is a bit alarming as many studies have reported adverse drug reactions when herbal medicines are taken concurrently with conventional medicines [29]. Another study stated that 64% of patients previously utilised herbs to manage diabetes; among them 27.0, 20.3, 15.2, and 10.8% used myrrh, black seeds, fenugreek and aloe respectively. 54.2% of respondents in this study did not experience any negative effects with herbs; 64.5% saw an improvement in blood sugar levels with herbs [30].

With respect to the association between demographics and knowledge regarding herbal medicines, some strong associations were found with respect to income, educational status, and gender. Respondents with higher income (> 5000 or 10,000 Saudi Arabia Riyals) were found to be significantly associated with the statement that “Herbal medicines can prevent all diseases”. However, majority of the participants (318 out of 409) fell under the category of low salary (< 5000 Saudi Arabia Riyals). Hence, this finding may not be a true representative of the general population and may need comparable number of participants in each category to ensure this outcome. A recent study conducted by University of Hail among general public in the Northern part of Saudi Arabia stated that the participants with higher income were significantly associated with more knowledge towards herbal medicines [20]. Regarding association with educational status, significant association with two statements of knowledge with an increase in educational status was found, that is statement 3 “Herbal medicines are preferred because of less side effects” and statement 7 “Overuse of herbal medicines can cause adverse effects”. This is in line with previous studies that higher education impacts on the knowledge of respondents [20]. Moreover, the impact of education on increasing awareness about adverse side effects due to overuse (statement 7 as mentioned above) is encouraging because overuse of anything including herbal medicines can be deleterious to health. With respect to gender, only statement 9 “Herbal medicines don’t need consultation with doctors” was found to be significantly associated with male gender who agreed with this statement. It is encouraging to see that although females are significantly associated with more use of herbal medicines (Table 2), they still think that herbal medicines need consultation with doctors. This creates a need to direct more awareness programs towards male population of the society.

In terms of attitude, results are slightly contradictory and confusing. Majority of the respondents looked optimistic about using herbal medicines than conventional medications that are prescribed by physicians. On the other hand, a minority of the people thought that herbal medicines are more effective than conventional medicines. These findings reflected that responses lacked clarity of information and supported the findings of previous studies that there is a lack of proper knowledge about herbal medicines among population [31, 32]. Moreover, with respect to attitude, 45% agreed that herbal medicines are safe which is slightly lower as compared to 61% in Kuwait population; it may be due to lower sample size in our study [22]. Approximately, more than half of the respondents in our study believed that herbal medicines can be used to improve and maintain health and treat illnesses which is similar to that in Kuwait, Canada and Australia [22, 33]. This could be explained by a general perception that these herbs are natural, hence will result in good effective outcomes for their health. With respect to association of demographics with attitude towards herbal medicines, only educational status and income of the respondents were found to be significant. Again, acquiring higher education is significantly associated with statement 1 “Herbal medicines can be used to help maintain and promote health” as compared to those with either no or less education. This may be due to the observation that literate people are more concerned towards maintaining their health as they are exposed to more sources of information, including books, newspapers, media etc. It is well documented that people with low literacy not only tend to delay treatment and diagnosis, but are also associated with increased mortality risk [34]. The same statement 1 is also significantly associated with increase in income. This may be due to the observation that people with less income are more focused to fulfil their basic daily necessities rather than spending much on promotion and maintenance of health. Furthermore, low income can further deteriorate this scenario by causing other hindrances like difficulty to access healthcare, lack of proper education etc. [35].

Regarding practices towards herbal medicines, results are quite interesting and convincing. Most strikingly, respondents (49.9%) agreed to use herbal medicines as a first choice when they get sick. This reflects the extent of practices towards herbal medicines by the general public in the second largest city of Saudi Arabia. Our findings are in line with the previous study which documented that more than half of the participants (54%) used herbal medicines as a first line therapy in Northern Saudi Arabia [20]. This may be because herbal medicines are generally considered safe and readily available. Another important finding is the use of herbal medicines without consultation of doctors by majority of the participants. Unsupervised use of herbal medicines can be dangerous in some circumstances where people are on conventional drugs, increasing the chances of drug-herb interaction and may lead to adverse events [36]. This calls upon the need for government authorities to conduct awareness campaigns in public regarding the proper use of herbal medicines, especially in case of concomitant use of conventional drugs. On the other hand, herbal medicine practitioners in Saudi Arabia should also be encouraged to incorporate scientific evidence-based practice (EBP), which seems to be quite low compared to herbal medicine practitioners in Western countries [37]. Similar recommendations were made by a study in Canada that both consumers and physicians should be kept updated about safety and efficacy of herbal products and also by registering herbal products as drugs [38]. A satisfying finding is the practice of around 43% of respondents towards looking at the expiry date of herbal medicines. In general, people believe that herbal medicines are safe and do not expire even after long time. However, this may result in lack of efficacy or sometimes side effects from degraded by-products within herbal medicines as with any other pharmaceutical products. Surprisingly, almost equal number of participants said yes and no to the statement, “I give herbal medicines to my children if they suffer from fever or pain” i.e. 33 and 33.7% respectively. Although, herbal medicines are supposed to be safe and effective in many diseases, still opting them for children in acute conditions, like fever, might not be a suitable choice, especially without proper supervision of a qualified doctor. Studies have documented safety as well as several side effects of herbal medicines in children [39, 40]. Hence, special care is required while using herbal medicines in children and awareness programs/lectures arranged by hospitals or government agencies might be a good option to overcome this issue. With respect to association of demographics with practices of herbal medicines, only educational status and gender of the respondents were found to be significant (*p* < 0.05). Acquiring education from primary till university level gradually impacted practice statement 6 i.e. “I take herbal medicines according to the instructions on the label”. These are encouraging results and signify the importance of education in the ethical practice of herbal medicines. Furthermore, proper labelling of herbal medicines products with substantial details about the constituents, usage and plausible side effects may help in decreasing the adverse events among general public while consuming herbal medicines [41]. In terms of gender impact on the practice statement no. 7 i.e. “I always look at the expiry date of herbal medicines before taking it”, female respondents practice it more than the males. Previous studies were not found to compare this result of the study, but this may be because females, in general, are more cautious about their appearance and expired products may cause adverse events like allergy on skin etc. However, females are found to be reluctant and rarely share the use as well as the side effects of herbal medicine with doctors. This calls for measures to decrease barriers in communication between female users and practitioners [42]. However, the practice of looking at the expiry date should be encouraged in both genders and commercials, as well awareness campaigns, can help in this regard.

Lastly, association of age was examined with knowledge, attitude and practice statements via ANOVA test and found significant associations only with two statements of knowledge i.e. “Herbal medicines are preferred because of few side effects” and “Overuse of herbal medicines can cause adverse effects”, and one statement of practice i.e. “I give herbal medicines to my children if they suffer from fever or pain”. Interestingly, increase in mean age was significantly associated with all three of the above statements indicating the impact of growing older on the knowledge and practice of herbal medicines. It is reported earlier that people with higher age are more inclined towards herbal medicines and hence their knowledge and practice toward herbal medicines also increases with the increasing age [43]. This may be due to the fact that people with higher age are consuming more medicines compared to younger age group to prevent diseases and maintain their health [44]. However, emphasis should be made to increase awareness regarding polypharmacy and drug-herb interaction, particularly in elderly, to avoid any detrimental events. It is highly recommended that the elderly population should be educated on the ethical practice of herbal medicines regarding its usage in children, particularly emphasizing on the need to consult with qualified traditional or allopathic doctors. Use of herbal medicine among Saudi population is a common practice and is related with female gender, adult population, and low level of education [45], which is also evident from the findings of this study. A recent study by Alghethmi et al. explored the specific role of gender in the use of herbal medicnes and found female gender to be more aware about herbal medicines and this knowledge increases with age [46]. Herbal medicines have been used and are still widely used in Saudi Arabia and other countries (Uganda) in treating and preventing diseases as well as during pregnancy [1, 21, 47]. This is mainly because users believe that herbal medicines are safe and without side effects. However, plants and herbal medicines are extremely complex material which are not devoid of adverse effects. In addition, there is lack of protocols and suitable methods of evaluating the products which is another barrier in the scientific validation of the effectiveness as well as adverse effects of these medicines. Herbal medicinal products need to be regulated by drug regulatory authorities similar to synthetic drugs to enhance quality, safety, and efficacy [48].

## Conclusion

In conclusion, the use of herbal medicines is quite common among the general population of Jeddah, Saudi Arabia. Participants were of the view that herbal medicines can be taken with conventional or allopathic medicines and can also be given to children without consultation with doctors or pharmacists; this is an alarming concern. Although most of the participants believed that the herbal medicines are safe, however, there is a dire need to establish effective strategies to evaluate the safety, efficacy, and quality of herbal medicines. Finally, the study covered in detail all three important aspects of KAP (Knowledge, Attitude, and Practices) regarding herbal medicines and demonstrated associations with different demographic categories. However, more studies are required locally, as well as globally, and there is a dire need to design and implement awareness programs both for the general public as well as for health-related professionals.

## Supplementary Information

**Additional file 1.** Questionnaire of the study (English version). File contains all the questions asked from the participants during the study. There are 5 parts of the questionnaire asking about demographics, and usage, knowledge, attitude, and practice of herbal medicine.

## Data Availability

The datasets used and/or analyzed during the current study are available from the corresponding author on reasonable request.

## Abbreviations

IRB: Institutional Review Board
KAIMRC: King Abdullah International Medical Research Center
WHO: World Health Organization
OTC: Over the Counter
CAM: Complementary or Alternative Mdicine
AIDS: Acquired Imunodeficiency Syndrome
HIV: Human Immunodeficiency Virus
SPSS: Statistical Package for the Social Sciences
IBM: International Business Machines
KAP: Knowledge, Attitude, and Practices
