# Supplementary material for: Public knowledge, attitudes, and practices towards herbal medicines; a cross-sectional study in Western Saudi Arabia
Source: BMC Complement Med Ther. 2022 Dec 8;22:326. doi: 10.1186/s12906-022-03783-y (PMC9733054; doi:10.1186/s12906-022-03783-y)
Supplement: Supplementary file 1 — Additional file 1. Questionnaire of the study (English version). File contains all the questions asked from the participants during the study. There are 5 parts of the questionnaire asking about demographics, and usage, knowledge, attitude, and practice of herbal medicine. [file 12906_2022_3783_MOESM1_ESM.pdf]

Kingdom of Saudi Arabia
King Saud bin Abdulaziz University
for Health Sciences

المملكة العربية السعودية
جامعة الملك سعود بن عبدالعزيز
للعلوم الصحية

A cross sectional survey on general public knowledge, attitudes and practices towards herbal medicines usage

Part 1: Demographic Characteristics:

1- Age: .... years old

2- Gender: ☐ Male ☐ Female

3- Ethnic background: ☐ Saudi ☐ Egyption ☐ Jordanian ☐ Pakistani ☐ Other (.......................)

4- Marital status: ☐ Single ☐ Married

☐ Widowed ☐ Divorced ☐ Separated

5- Highest Educational Status:
☐ Primary School ☐ Secondary School ☐ College ☐ University ☐ None

6- Employment Status:
☐ Employed for wages ☐ Self employed ☐ Housewife ☐ Retired ☐ Unemployed

7- Monthly Income: ☐ <3000 SR ☐ 3000 SR ☐ 5000 SR ☐ 10,000 SR ☐ >10,000 SR

8- Occupation related to health care:
☐ Yes ☐ No

9- Family member's occupation related to healthcare:
☐ Yes ☐ No

10- Cigarette Smoking:
☐ Yes ☐ No

11- Shisha Smoking:
☐ Yes ☐ No

12- Please select any of the following chronic medical condition, which you are currently having? (can choose more than one answer)

☐ Respiratory illness ☐ Diabetes ☐ Hypertension ☐ Heart problems

☐ Kidney diseases ☐ Joint diseases ☐ None ☐ others: ....

13- Number of visit to herbal store in the last 12 months:

☐ 1-4 times ☐ 05-10 times ☐ >10 times ☐ None

14- Personal health:

☐ Excellent ☐ Very good ☐ Good ☐ Fair ☐ Poor

Part 2: Usage of Herbal Medicines

1- In the past 6 month, have you taken any herbal medicine? (If your answer is YES. please proceed to the following question if your answer is NO, please proceed to Part 3**)**

☐ Yes ☐ No

2- Source of herbal medicine taken is from (you may choose more than one)**:**

☐ Prescribed and given by hospital clinic after consultation

☐ Purchased from a retail pharmacy

☐ Purchased from a herbal store without consultation with doctor

☐ Use from someone's herbal medicine

☐ Others:..(please specify)

3- Please select the reason you take the herbal medicine. (can choose more than one answer)**:**

☐ Respiratory illness ☐ Urinary tract infection

☐ Abdominal pain ☐ Dental pain

☐ Skin problem or wound ☐ Joint diseases

☐ Hypertension ☐ Diabetes

☐ Others:..(please specify)

Part 3: Knowledge of Herbal Medicines

No.	Statement	YES	NO	NOT SURE
1.	Herbal medicines are made from plant source			
2.	Herbal medicines can be from animal source			
3.	Herbal medicines can prevent all diseases			
4.	Herbal medicines can cure all diseases			
5.	Herbal medicines are preferred because of less side effects			
6.	Herbal medicine is always safe			
7.	Overuse of herbal medicine can cause adverse effect			
8.	Herbal medicines can be taken with conventional or allopathic medicines			
9.	Herbal medicines don't need consultation with doctors			
10.	Herbal medicines don't expire			

Part 4: Attitude Towards Herbal Medicines

NO.	Statement	Strongly agree	Agree	Neutral	Disagree	Strongly disagree
1.	Herbal medicines can be used to help maintain and promote health					
2.	Herbal medicines can be used to treat illness					
3.	Herbal medicines are safe because they are made from natural ingredients					
4.	Herbal medicines are better for me than Conventional or allopathic medicines					
5.	A lot of the health claims made by the manufacturers of Herbal medicines are unproven					
6.	I don't feel that Herbal medicines are dangerous for children					
7.	I prefer Herbal medicines because they are cheap and easily available					
8.	It is important to talk to a medical doctor or herbal doctor or a pharmacist before using Herbal medicines					

Part 5: Practices Towards Herbal Medicines Usage

NO.	Statement	Strongly agree	Agree	Not sure	Disagree	Strongly disagree
1.	When get sick, I first take Herbal medi-cines to help me get better					
2.	I do not consult doctors before taking Herbal medicines					
3.	I also give Herbal medicines to my family members if they get sick					
4.	I take Herbal medicines in case of acute conditions like severe pain					
5.	I give Herbal medicines to my children if they suffer from fever or pain					
6.	I take Herbal medicines according to the instruction on the label					
7.	I always look at the expiry date of Herbal .medicines before taking it					
8.	I advise others to take Herbal medicines whenever they have problems					

Thank you for your participation
